# Effect of Blood on Synovial Joint Tissues: Potential Role of Ferroptosis

**DOI:** 10.3390/app14146292

**Published:** 2024-07-19

**Authors:** Howard J. Nicholson, Neeraj Sakhrani, James Rogot, Andy J. Lee, Inioluwa G. Ojediran, Ratna Sharma, Nadeen O. Chahine, Gerard A. Ateshian, Roshan P. Shah, Clark T. Hung

**Affiliations:** 1 Department of Biomedical Engineering, Columbia University, New York, NY 10027, USA; 2 Department of Orthopedic Surgery, Columbia University, New York, NY 10032, USA; 3 Department of Mechanical Engineering, Columbia University, New York, NY 10027, USA

**Keywords:** knee, blood, hemarthrosis, hemophilia, synovitis, fibrosis, ferroptosis, osteoarthritis

## Abstract

Recurrent bleeding in the synovial joint, such as the knee, can give rise to chronic synovitis and degenerative arthritis, which are major causes of morbidity. Whereas chronic arthropathy affects one-fifth of hemophiliacs, conditions such as rheumatoid arthritis (RA), periarticular and articular fractures, osteochondral autograft transplantation surgery, and anterior cruciate ligament (ACL) injury are also associated with joint bleeding. Synovial joint trauma is associated with inflammation, acute pain, bloody joint effusion, and knee instability. Clinically, some physicians have advocated for blood aspiration from the joint post-injury to mitigate the harmful effects of bleeding. Despite the significant potential clinical impact of joint bleeding, the mechanism(s) by which joint bleeding, acute or microbleeds, leads to deleterious changes to the synovial joint remains understudied. This review will address the impact of blood on synovial joint tissues observed from in vitro and in vivo studies. While the deleterious effects of blood on cartilage and synovium are well-described, there are much fewer reports describing the negative effects of blood on the meniscus, cruciate ligaments, and subchondral bone. Based on our studies of blood in co-culture with chondrocytes/cartilage, we raise the possibility that ferroptosis, an iron-dependent, nonapoptotic form of regulated cell death, plays a contributing role in mediating hemophilic arthropathy (HA) and may represent a therapeutic target in reducing the negative impact of joint bleeds.

## 1. Introduction

Synovial joints allow for a significant range of motion, including the flexion and extension of the knee, and are characterized by a synovial cavity that contains synovial fluid (SF) [1]. This viscous solution is responsible for joint lubrication and reducing friction between articular cartilage during movement. Under arthritic conditions, the rheological properties of synovial fluid are significantly impaired [2]. Synovial neutrophils from patients with rheumatoid arthritis (RA) and osteoarthritis (OA) produce significantly higher levels of reactive oxygen species [3]. Increased inflammation and oxidation stress exacerbate the degradation of hyaluronic acid (HA), a major component in SF, leading to various joint-related diseases [1].

**Figure 1.**
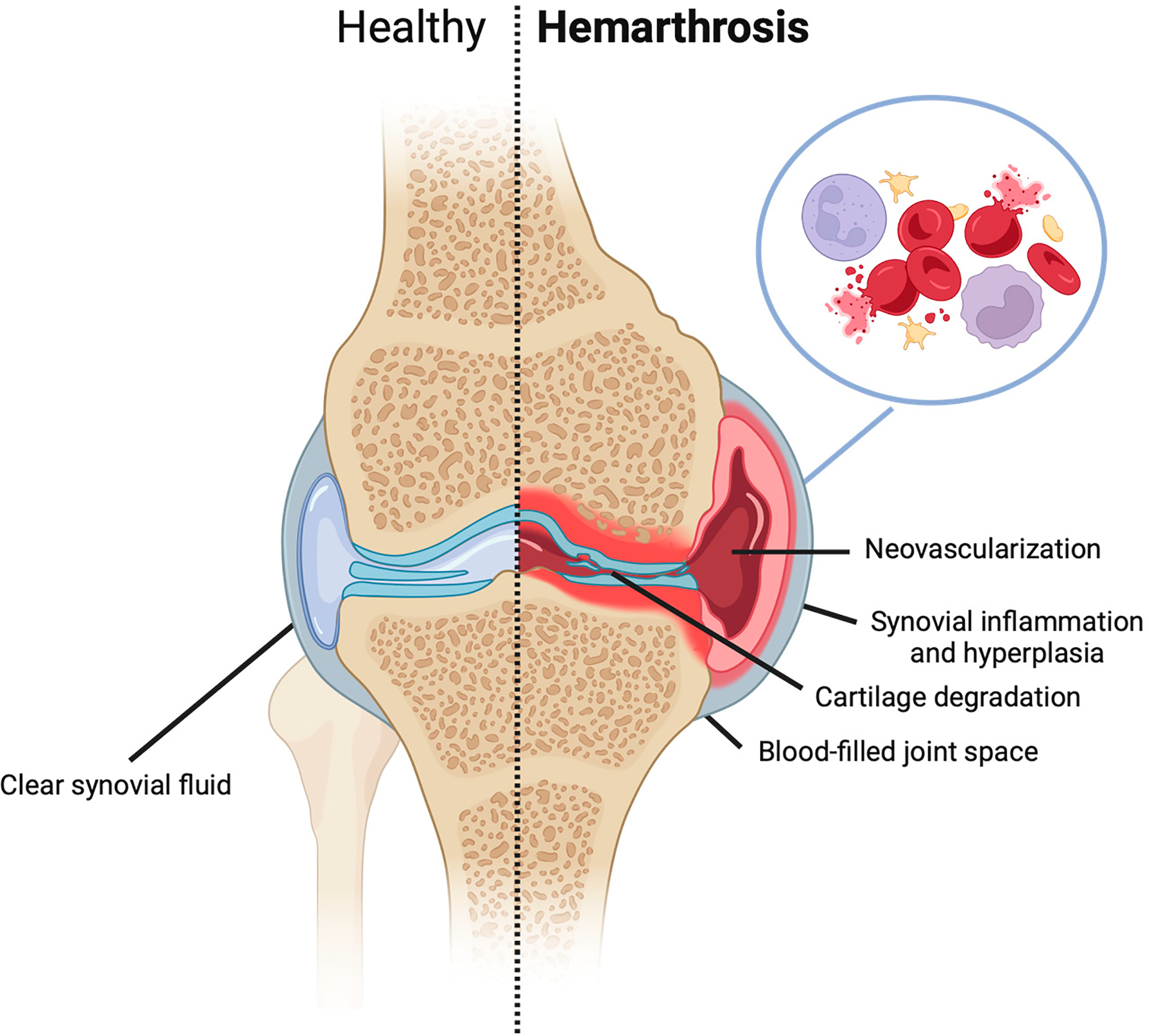
Human knee with and without hemophilia-induced bleeding and inflammation into the joint space after traumatic injury.

Joint problems resulting from recurrent joint bleeding or hemarthrosis, such as chronic synovitis and degenerative arthritis, are major causes of morbidity, where chronic arthropathy affects ~20% of hemophiliacs [4]. In addition to hemophilia, conditions such as rheumatoid arthritis (RA), periarticular and articular fractures, osteochondral autograft transplantation surgery, and anterior cruciate ligament (ACL) injury are associated with joint bleeding [4–8]. Additionally, traumatic injuries to the synovial joint have been associated with inflammation, acute pain, bloody joint effusion, and knee instability (Figure 1). To curtail the deleterious effects of blood, some clinicians recommend aspiration of blood from the joint post-injury and prior to surgical closing [8,9]. Despite the significant potential clinical impact of joint bleeding, the mechanism(s) by which joint bleeding, acute or microbleeds, leads to deleterious changes to the synovial joint remains understudied [7]. While apoptosis is a primary candidate for regulated cell death (RCD) in blood-exposed chondrocytes, based on our studies of blood in co-culture with chondrocytes/cartilage (as mentioned in Section 4.1), we speculate that an iron-dependent, nonapoptotic form of RCD, ferroptosis, plays a critical role in the development of hemophilic arthropathy (HA) and may impact injurious changes to joint tissues [10–14]. While the damaging effects of blood on cartilage and synovium are well-described, there are much fewer reports describing the negative effects of blood on the meniscus, cruciate ligaments, and subchondral bone [15–19]. This paper aims to provide a comprehensive overview of the impact of blood on synovial joint tissues from clinical observations as well as in vitro and in vivo investigations. A better understanding of blood–synovial tissue interactions may help to inform the development of therapeutic treatments that can mitigate the deleterious effects of synovial joint bleeding.

## 2. The Etiology of Hemarthrosis

A main characteristic of hemarthrosis includes iron deposition in both synovial and subsynovial tissues, accompanied by neutrophil and macrophage infiltration of these tissues [20]. Blood exposure and oxidative stress due to hemophilic arthropathy or trauma can alter the synovial fluid environment and induce the release of pro-inflammatory cytokines (ex. IL-6, IL-1β, TNFα, IL-4, and IL-10) [21]. Recurrent bleeding increases the potential for red blood cell (RBC) lysis, further exposing the joint to elevated levels of iron following hemoglobin release. Furthermore, excess iron deposition can trigger synovial proliferation and inflammation or synovitis, which activates the immune system, leading to synovitis and angiogenesis (Figure 1) [20]. Consequently, the inflamed synovium is now at risk of repeated injury and rebleeding, leading to macrophage-like synoviocytes releasing pro-inflammatory cytokines and matrix metalloproteinases, while fibroblast-like synoviocytes exhibit uncontrolled expansion and thickening [22]. As a result, joint cartilage becomes degraded by the new invasive synovial growth [23]. Research is ongoing to ascertain whether synovial changes due to blood-induced joint damage are independent of cartilage degradation [24].

Hemarthrosis can be attributed to traumatic or nontraumatic etiologies, involving various pathological mechanisms that expose blood to the joint space [8]. Acute injuries resulting in abnormal twisting of the loaded joint often contribute to hemarthrosis [25]. Specifically, ligament ruptures, articular fractures, patellar dislocations, meniscal tears, and osteochondral injuries are common traumatic events associated with bleeding [8,26]. In a recent study, hemarthrosis was observed in 98% of patients with acute knee injuries [27]. The blood that enters the joint as a result of these conditions will reach up to 100% *v*/*v* and is typically aspirated within a week if bleeding stops, compromising the structural and functional properties of surrounding tissues over time [10].

Among these injuries, partial or complete ACL tears are the predominant cause of intra-articular bleeding [15]. In a recent study, 77% of cases of acute hemarthrosis were caused by ACL ruptures [28]. Further studies have shown that within 4 h of trauma-induced hemarthrosis, significant degradative effects were observed in neighboring tissues including the cartilage, meniscus, and bone [29]. Damage to the joint is often accompanied by an increase in pro-inflammatory cytokine levels in the surrounding synovial fluid, indicating that these biomarkers may stem from intra-articular bleeding episodes [27]. Specifically, it has been shown that injured patients with hemarthrosis exhibit elevated IL-1β, TNF-α, IL-6, IL-8, MMP-3, and TIMP-1 levels in the SF post-injury [30–32]. In addition to synovial inflammatory cytokines, ECM degradative products also increase upon injury [27]. Of note, patients suffering from these traumatic incidents were observed to have a 50% higher likelihood of developing posttraumatic osteoarthritis (PTOA) within 15 years of initial injury [33]. However, precise factors contributing to PTOA pathogenesis following joint injury and concurrent hemarthrosis continue to be insufficiently explored. Unlike OA whose etiology is not well known, the initiation of PTOA is well defined, which provides an opportunity for therapeutic intervention such as a disease-modifying osteoarthritic drug (DMOAD) that will disrupt the subsequential development of OA.

A subset of traumatic hemarthrosis involves an injury resulting from minimal trauma. In the majority of cases, the occurrence of such injuries may result in underlying bleeding diathesis [8,34]. Postoperative hemarthrosis may also occur following arthroscopic procedures, although the incidence is typically low [8,35]. A study examining 8500 arthroscopic surgeries conducted over 13 years reported only three cases of hemarthrosis [35]. Lateral retinacular release surgery was associated with the highest complication rate, primarily due to the risk of bleeding from injury to the superior lateral genicular artery during the procedure [35,36]. In adults, hemarthrosis can also arise as a complication of total knee arthroplasty, but it occurs in fewer than two percent of patients undergoing surgery [37]. These unsuccessful procedures can result in gradual pain and elevated inflammation within months post-surgery.

Hemarthrosis can also occur spontaneously as a result of congenital bleeding disorders like hemophilia [15,38]. An inherited X-linked recessive condition, hemophilia affects approximately 1 in 5000 males [38]. The deficiency of coagulation factor VIII or IX in hemophilia leads to recurring spontaneous bleeds, often causing contractures or degenerative conditions in severely impacted joints [4,39]. The initial occurrence of hemophilia typically manifests in children during the early years, with the synovium being a highly affected structure [8,38]. Typically, the synovium functions to uptake foreign substances and evacuate debris from the joint cavity following a traumatic injury [15,40]. However, with recurrent joint bleeds, the synovium’s ability to remove foreign materials is impaired, resulting in the onset and progression of synovitis [15,41]. Hemophilic synovitis can develop further into HA [39,42]. With recurrent bleeding episodes, HA gradually contributes to debilitating pain, joint deformity, limited movement, and loss of function [43]. Despite the availability of clotting factor replacement therapy, about 20% of patients continue to experience chronic HA due to repeated intra-articular bleeding episodes [44].

## 3. Interplay of Erythrocytes, Hemoglobin/Iron, and Synovium

Intact erythrocytes can communicate with other cell types via chemical signaling as they are known to be reservoirs of chemokines. The median concentration of cytokines, chemokines, and growth factors in RBCs is 12-fold higher than in the plasma [45]. RBC responses to shear can modulate its secretome, such as the increase in endothelial nitric oxide synthase [46]. Erythrophagocytosis resulting in lysis of RBCs by synovial cells occurs in culture, during which synovial cells absorb hemoglobin from the culture medium within 4 h of blood introduction and convert the iron to ferritin [47,48]. Ferric iron may either be reduced to the ferrous form and promote the formation of toxic-free radical species, or stimulate collagenase and prostaglandin release from synovial macrophages [49,50]. Synovial cells, and in particular the Type A or macrophage-lining cells, have been reported to have a strong affinity for iron [51]. While blood itself may not persist in the joint space, the remnants of erythrocytes including hemoglobin and deposited iron (hemosiderosis) may have longer-lasting or permanent effects as heme interacts with immune and synovial cells to induce the production of pro-inflammatory cytokines (e.g., IL-1 and TNFα) as well as fibroblast proliferation leading to tissue hyperplasia [49,52]. Researchers have shown that following hemarthrosis, patients exhibited an increase in pro-inflammatory cytokines in their SF 24 h after injury, followed by a time-dependent decrease [53].

### 3.1. Oxidative Stress

**Figure 2.**
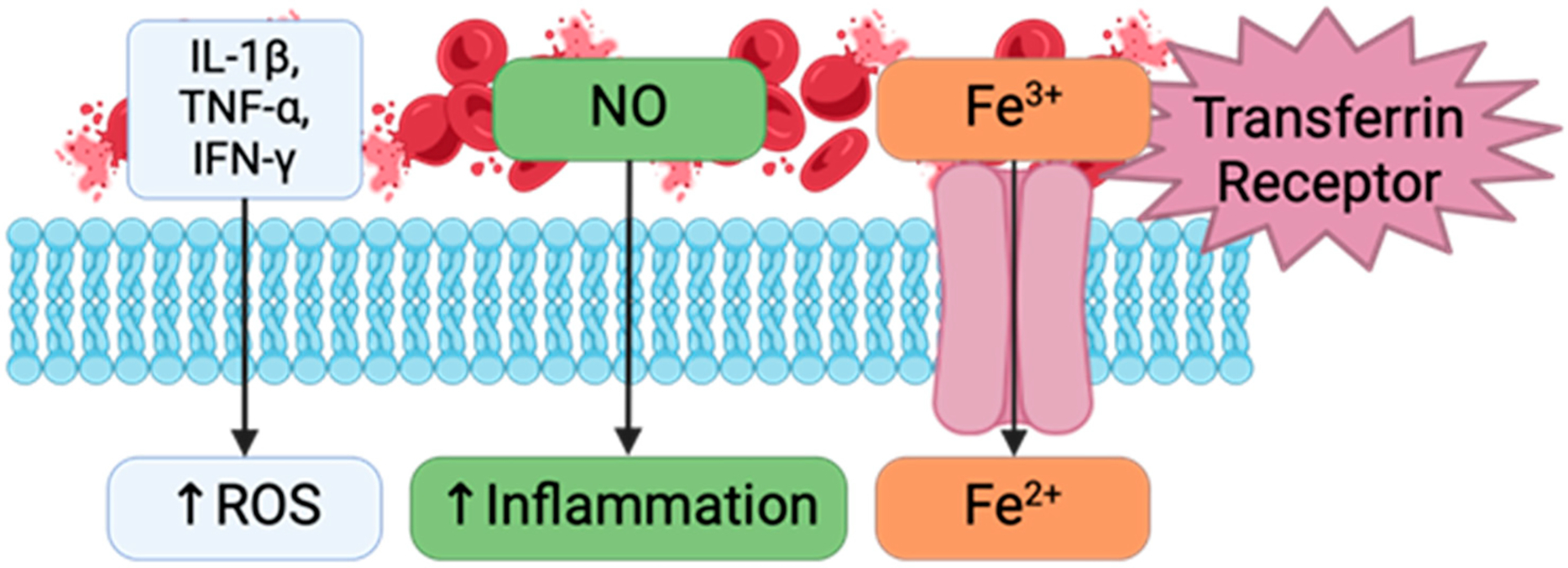
Inflammatory pathway highlighting the effect of red blood cell lysis and iron exposure on oxidative stress and cytokine release.

Following blood exposure, the joint is susceptible to iron-generated reactive oxygen species (ROS) from hemoglobin. This can lead to lipid peroxidation and the inactivation of glutathione peroxidase 4, which has been shown to contribute to OA progression [14]. The presence of lipid peroxidation end-products in the synovial fluid of OA patients and their catabolic effects on cartilage ECM has previously been documented [54]. Blood components have been directly linked to ROS-mediate and ferroptotic mechanisms that contribute to the phenotype of HA. In addition to lipid peroxidation, researchers have observed increases in mitochondrial ROS expression following iron and blood treatment indicating that blood exposure and iron deposition can induce oxidative stress (Figure 2) [14].

### 3.2. Role of Iron

Ferrokinetic studies with radiolabeled RBCs suggest microbleeding as a contributory factor where iron is a major catalyst in the production of hydroxyl radicals, which can induce considerable tissue damage, degrade synovial fluid hyaluronan, and attack the articular surface [5]. Erythrophagocytosis resulting in lysis of RBCs by synovial cells occurs in culture, during which synovial cells absorb hemoglobin from the culture medium within 4 h of blood introduction and convert the iron to ferritin [47,48]. Ferric iron may either be reduced to the ferrous form and promote the formation of toxic-free radical species, or stimulate collagenase and prostaglandin release from synovial macrophages [49,50]. Synovial cells, and in particular the Type A or macrophage-lining cells, have been reported to have a strong affinity for iron [51]. Blood-injured cartilage has also been reported to be less capable to withstand loading in vivo [53,55].

### 3.3. Ferroptosis Pathway

**Figure 3.**
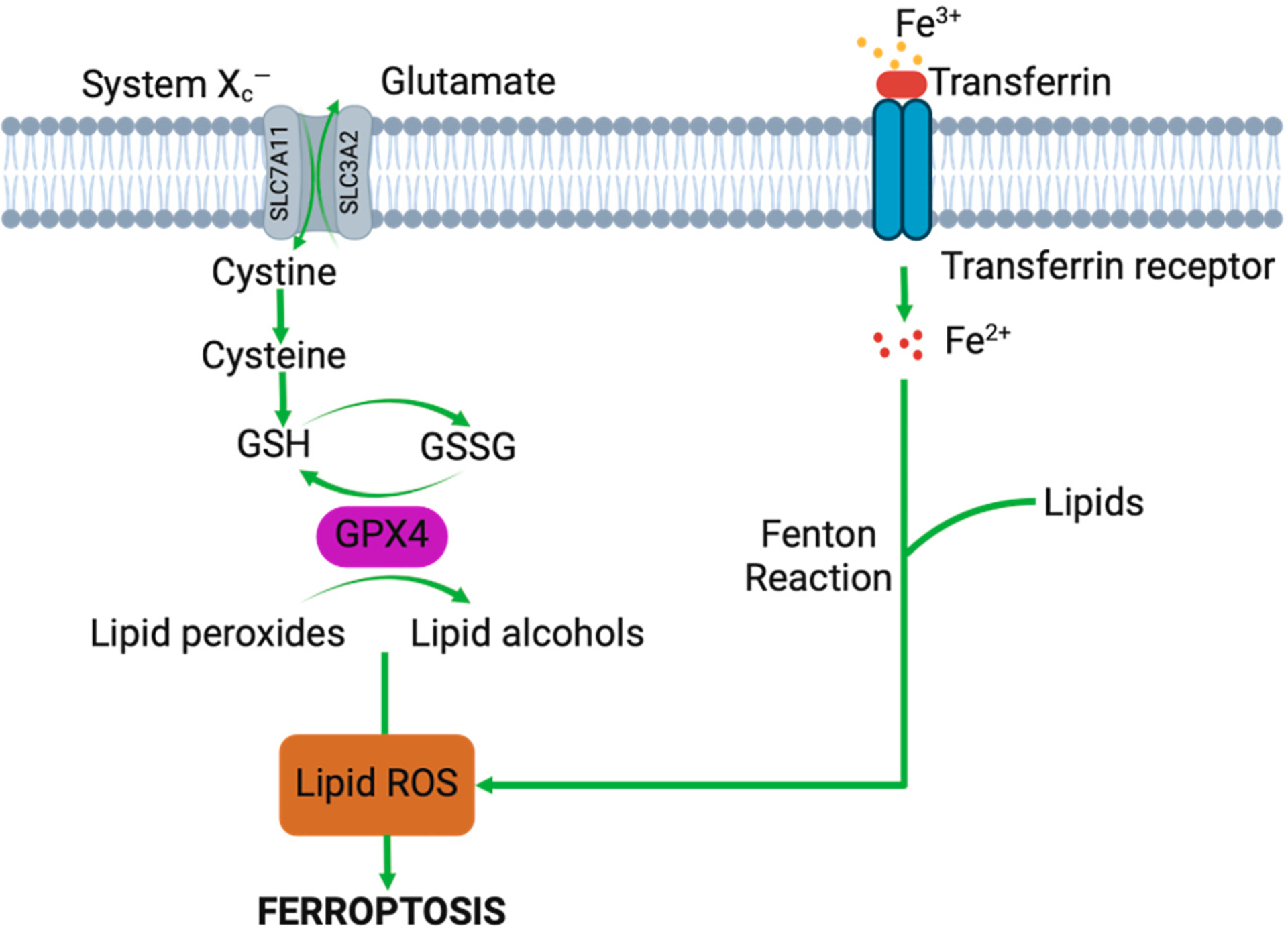
GPX4 pathway showing implications of iron deposition on intracellular lipid ROS production and downstream ferroptosis-induced cell death.

Ferroptosis is associated with excessive intracellular accumulation of lipid hydroperoxides formed from free hydroxyl radicals and polyunsaturated fatty acids (Figure 3) [11,13]. Specifically, intracellular free radicals such as hydrogen peroxide undergo a Fenton reaction with imported Fe^2+^ to form harmful hydroxyl radicals that spontaneously react with polyunsaturated fatty acids to form lipid hydroperoxides. Glutathione peroxidase 4 (GPX4) normally converts harmful lipid hydroperoxides to harmless lipid alcohols through parallel conversion of glutathione to glutathione disulfide. Intracellular glutathione supply is dependent on a cysteine/glutamate antiporter, System X_c_^−^. As such, inducers of ferroptosis include inhibitors of System X_c_^−^ (e.g., erastin, high extracellular glutamate) as well as those of GPX4 function (e.g., (1S,3R)-RSL3, (RSL-3)). Compounds that counter ferroptosis include those that prevent lipid hydroperoxide formation by radical trapping (e.g., Fer-1 and Liproxstatin-1) [56–58]. Hemarthrosis and iron release can trigger this relatively newly defined cell death pathway. Evidence of ferroptotic induction following blood exposure has been shown in vitro and in vivo and usage of ferroptotic inhibitors has shown promise in mitigating blood-induced deleterious effects [11,13,14,59,60]. Pro-inflammatory factors like nitric oxide and various interleukins can also trigger the ferroptosis pathway [21,45].

### 3.4. Hemarthrosis-Induced Cytokines

Blood can directly affect articular chondrocytes and cartilage by inducing apoptosis, the long-term suppression of proteoglycan synthesis, and the reduction in mechanical properties [1,12,24,55,61]. In human blood, the WBC fraction will include blood-derived monocytes in culture as they are the dominant immune cell in HA and transition to macrophages upon synovial tissue interactions where resident synovial fibroblasts exhibit plasticity and can exhibit macrophage-like markers when subjected to pro-inflammatory cytokines that are capable of erythrophagocytosis [24,49,62,63]. Preliminary data have demonstrated the deleterious effects of blood and the mediating role of ferroptotic mechanisms on human-derived engineered cartilage, chondrocyte monolayers, and native explant tissues, as well as ACL fibroblast 2D and 3D cultures.

### 3.5. Fibrosis

In addition to inducing ferroptosis, blood exposure can induce alpha-smooth muscle actin (α-SMA) expression, indicating the transition of fibroblast-like synoviocytes (FLS) to a myofibroblast phenotype (fibrosis) by blood [64,65]. In the context of wound healing, the cell is attempting to repair an injury and restore function. Within a short time span, various immune cells are drawn from the blood that can release factors that influence neighboring cell behavior [64]. Chronic wounds or trauma can induce persistent inflammation, which can cause the wound-healing process to fail [65]. These cells become activated to generate further pro-inflammatory cytokines, thereby initiating an inflammatory cascade that may lead to fibrosis [66]. This can manifest via the accumulation of excess matrix or scarring.

### 3.6. Role of Blood Components

In addition to erythrocytes, the various components of whole blood are implicated in the trauma and inflammatory response of the synovial joint. For instance, the fraction of white blood cells (WBCs) in whole blood also includes blood-derived monocytes in culture as they are the dominant immune cell in hemophilic arthropathy and transition to macrophages upon synovial tissue interactions, where resident synovial fibroblasts exhibit plasticity and can exhibit macrophage-like markers when subjected to pro-inflammatory cytokines, which are capable of erythrophagocytosis [24,49,62,63]. Additionally, platelets play an essential role in the wound-healing process. The majority of the literature elucidating the role of blood components in the synovial joint is limited to two-dimensional culture techniques. In recent years, a three-dimensional (3D) study measured the effects of various blood components using a collagen scaffold with an ECM composition more similar to the provisional scaffolds found in wound sites [67]. Researchers found that while erythrocytes inhibited proliferation and induced cell death, increased platelet concentration significantly affected contractility, proliferation, and cytokine release, which could affect regenerative potential [17].

## 4. In Vitro Models

Much about blood effects on synovial joint tissues has been gleaned from tissue culture studies of 2D and 3D cultures of isolated cells or tissue explants with applied physiologic loading and modern bioengineering and biology tools. These devised systems permit a systematic approach for a myriad of experimental conditions, including blood concentration and duration of application, and provide opportunities for mechanistic studies. Isolating the contribution of synovium-dependent and independent blood effects on joint tissues that share synovial fluid, as well as identifying anti-ferroptosis agents may help to define the role of bleeding in joint damage [68,69]. Current research has not investigated blood injury of the cartilage, bone, meniscus, and ligament in co-culture with synovium and/or under physiologic loading conditions in culture. There also are no established models for recurrent microbleeds in 2D in vitro models. To gain mechanistic insights into the role of individual blood constituents, whole blood, RBCs and lysed RBCs (such as occurs via phagocytosis by macrophages), WBCs (buffy coat), and platelets will need to be further studied [45,47,48,70].

In culture experiments, the concentration of blood stays constant, whereas, in situ, the synovial joint concentration decreases due to blood clearance. The majority of published studies of blood and joint tissues have been undertaken in a similar manner, which has also led to the development of a robust culture protocol for studying blood-induced injury of human cartilage to characterize blood-induced cell death, particularly ferroptosis, and testing the efficacy of anti-ferroptosis agents on preserving synovial joint health (Table 1) [71]. The contribution of ferroptosis to the co-culture of the cartilage, bone, meniscus, and ligament with synovium and applied loading will provide new mechanistic insights as well as potential therapeutic targets to arrest hemarthrosis. Establishing in vitro model systems is necessary to capture critical aspects of the inflammatory joint environment that may drive bleeding-induced synovial arthropathy.

### 4.1. Cartilage

**Figure 4.**
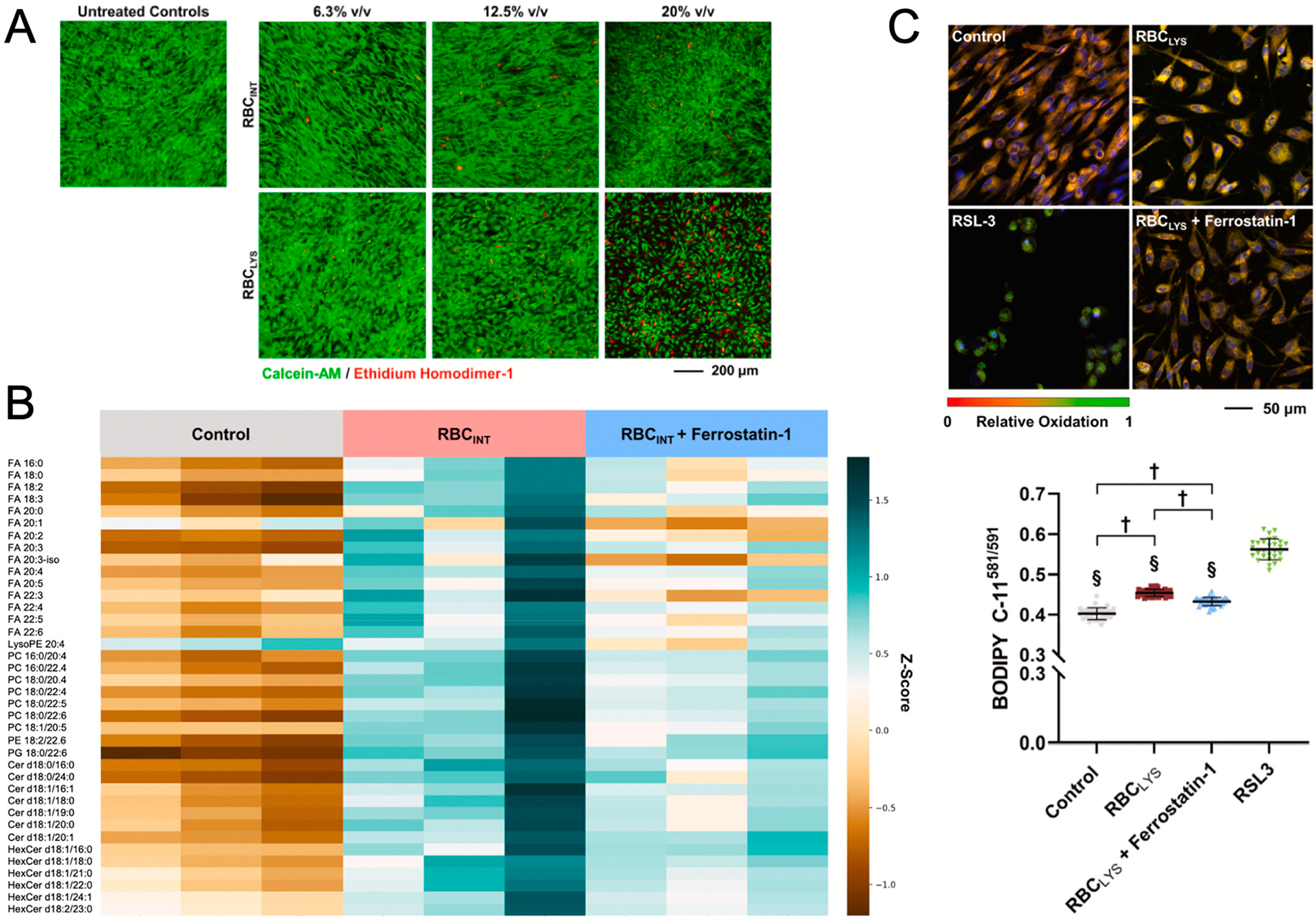
(**A**) Confocal imaging assessment of RBC-induced changes to chondrocyte viability following treatment with increasing concentrations of intact (RBC_INT_) and lysed (RBC_LYS_) erythrocytes. (**B**) Heatmap of significantly elevated lipid species in human chondrocytes in response to RBC_INT_ treatment and mitigation by Ferrostatin-1. (Row: detected lipid species, column: a sample). The relative abundance of each lipid is color-coded, with blue as high and brown as low signal intensity. (**C**) Lipid peroxidation induced by RBCLYS measured by the BODIPY C-11581/591 probe using the ferroptosis inducer, RSL3, as a positive control. The ratios of the intensity of oxidized undecanoic acid (Ex/Em: 488/510 nm) to the intensity of reduced undecanoic acid (Ex/Em: 581/591 nm) were measured for individual cells (N = 28–44 cells). † *p* < 0.0001. § *p* < 0.0001 vs. RSL3. (Adapted from Lee et al. [14]).

Blood has been shown to induce rapid and irreversible damage to cartilage that is characteristic of degeneration observed in the early stages of osteoarthritis (OA) [8,86–88]. Alarmingly, short exposure to a low amount of blood (10% *v*/*v* for 48 h) leads to prolonged and irreversible disturbances in cartilage matrix turnover still present ten weeks after initial blood exposure; the blood concentration in the synovial cavity reaches nearly 100% blood exposure; the blood concentration in the synovial cavity reaches nearly 100% during hemarthrosis episodes as a result of the small synovial fluid volume [10,52,61]. Recent studies by our group reported that intact RBCs induce intracellular phenotypic changes in chondrocytes that increase vulnerability to tissue damage while lysed red blood cells have a more direct influence on chondrocyte death (Figure 4A) by mechanisms that are representative of ferroptosis [14]. Specifically, increased lipid species (Figure 4B) and lipid peroxidation expression (Figure 4C) were observed in human chondrocytes treated with intact RBCs and RBC lysates (20% *v/v*) compared to the control, implicating the potential role of ferroptosis. Previous dose-response experiments have shown that 20% is the minimum blood concentration to induce significant cell toxicity, but physiological concentrations following injury can be even greater [14]. Moreover, the ferroptosis selective inhibitor ferrostatin (Fer-1) was observed to counter blood effects in chondrocytes/cartilage [14]. Our findings in cartilage research suggest the role of ferroptosis in mediating degenerative blood-induced cartilage changes that need to be further explored in other synovial tissue types.

### 4.2. Synovium

The synovium plays a critical role in maintaining the equilibrium between normal joint function and potential pathology. Joint trauma often leads to blood entering the joint space and mixing with synovial fluid (SF), thereby changing the rheological properties of SF and leading to increased iron deposition [77]. Also, synovitis and synovial angiogenesis are directly linked to OA progression and can exacerbate pain and joint damage [78]. Inflamed synovial tissue can often become hypoxic, which stimulates the production of hypoxia-inducible factor-1 (HIF-1) and vascular endothelial growth factor (VEGF), further promoting angiogenesis [89]. FLS also proliferate under inflammatory conditions, contributing to the growth and potentially invasiveness of new blood vessels. Researchers have also found that unspecified cells derived from human synovial tissue are able to synthesize ferritin, a storage form of iron, from the breakdown of erythrocytes and accumulation of iron deposits following hemarthrosis and the inflammation of synovial tissue [47]. This study highlights the degradative effects of erythrocytes found in whole blood but other blood constituents have also been shown to have restorative effects. To compare the efficacy of blood-derived products with other anti-inflammatory therapeutics, researchers treated unspecified cells derived from equine synovial fluid with platelet-rich plasma (PRP). While the addition of PRP did not increase ROS, it did increase concentrations of inflammatory cytokines up to 48 h after injection, leading them to conclude that PRP should be used with caution during the post-operative period [80].

### 4.3. Meniscus

The impact of hemarthrosis on the health of the meniscus, a fibrocartilaginous structure that aids in biomechanical functions, has also been studied. Although traumatic injuries to the meniscus are often accompanied by ACL trauma and bloody joint effusion, there is a lack of knowledge about the direct effects of blood on the meniscus. Notably, an in vitro study using a new femoral drill model on a rabbit to simulate hemarthrosis seen in ACL reconstruction (ACLR) observed a significant upregulation of inflammatory and catabolic gene expression [74]. The administration of dexamethasone (DEX) following surgery significantly decreased degradative and inflammatory markers in the rabbit meniscus [81]. Another study examined the effects of whole blood on porcine meniscus explants and on meniscal cells. Researchers found that whole blood exposure increased metalloproteinase (MMP) activity and nitric oxide production while decreasing GAG content [82]. Further research is needed to better understand the implications of blood exposure on meniscal healing.

### 4.4. Bone

In contrast to cartilage, meniscus, and ACL, bone is well-vascularized. Subchondral bone cysts in joints are also associated with hemophilia [19,90]. Bone biomarkers like osteopontin are typically not expressed in joint tissues other than bone, but high levels have been observed in OA cartilage [53]. The release of these matricellular proteins into the SF may induce an inflammatory response and lead to bone and cartilage turnover following joint trauma [53]. Joint deterioration following a knee injury with acute hemarthrosis has been observed in patients multiple years after the initial trauma. Characteristic radiographic changes following hemarthrosis included osteophyte formation and significantly increased bone uptake [91]. Intra-articular osteochondral fractures are typically accompanied by bleeding into the joint cavity and bony defects. Bone marrow stromal cells (BMSCs) are commonly used as a cell source for bone regeneration but are limited due to availability and the invasiveness of the retrieval process. After a joint fracture, a hematoma develops that releases a supply of BMSCs that are usually aspirated to treat pain and inflammation [92]. Scientists proposed that the fracture hematoma can be used as an alternative cell source for osteoprogenitor cells to treat bony defects. Researchers have since demonstrated the ability of human hemarthrosis-derived cells to differentiate into osteoblast-like cells and their potential to be utilized in bone regeneration therapy [83].

### 4.5. ACL

In terms of ACL reconstruction, researchers have begun to isolate components of blood that have regenerative potential. Martha Murray has performed studies to investigate the effects of peripheral blood mononuclear cells (PBMCs) and platelets on the behavior of ACL fibroblasts in a collagen scaffold [16,17]. The data suggest that the interaction of PBMCs and platelets leads to increases in collagen and IL-6 expression. Past results have shown the promise of platelet-rich plasma (PRP) as a therapeutic, but in combination with PBMCs, platelets, and a collagen scaffold, they could be used in ACL repair treatment [85]. Contrastingly, pilot data have highlighted the negative effects of certain blood constituents, particularly erythrocytes. For example, a gel contraction assay has been used as a 3D culture model to simulate the effect of blood constituent concentrations on the wound-healing process [67,71]. Murray found that increasing erythrocyte concentration limited scaffold contractility and significantly increased collagen production [16]. Ultimately, additional studies are needed to characterize the effects of blood constituents on ACL repair.

## 5. In Vivo Models of Hemarthrosis

Through a combination of existing approaches described for the in vivo study of animals in the literature, the interaction of joint bleeding (blood models) and wound repair can be studied. Despite gaps in research, there has been success in replicating hemarthrosis in various in vivo animal models, including mice, rats, rabbits, and dogs, via needle puncture or injection of blood into the synovial joint (Table 2) [93–96]. Blood can directly affect articular chondrocytes and cartilage by inducing apoptosis, the long-term suppression of proteoglycan synthesis, and the reduction in mechanical properties [12,52,55,61]. The injection of blood into synovial joints to replicate hemarthrosis in animals, including mice, rats, rabbits, and dogs, creates symptoms similar to those observed in hemophilic arthropathy [71,93–95]. Pigs and sheep are closer in anatomy and biomechanical properties to humans and have also been used in ligament repair studies. Although these animal models cannot be applied to all aspects of human anatomy and physiology, we can use multiple models for different purposes.

Strategies for cartilage repair are vast and each animal model has distinct advantages and limitations. For example, rats and rabbits have different cartilage healing abilities and gait and movement patterns, making them nonoptimal for comparison with humans [97]. Large animal models, like dogs and horses, address many of these discrepancies. The sizes of joints and articular cartilage, post-operative routines, and types of cartilage defects are more similar to humans in these large animal models [97]. However, because of the cost and ethical considerations associated with large animal use for research purposes, smaller animal models are more commonly used. Despite their drawbacks, small animal models are still useful for assessing therapeutics and devices, as well as testing variables and mechanisms associated with disease and injury models [97].

Mice and rats are most often utilized for genetic and chemical purposes and have established disease models and surgeries. They also provide the advantage of being easier to handle and suitable for microscopic observation [98]. The destabilization of medial meniscus (DMM)-induced OA mouse models also shows promise in studying the efficacy of therapeutic injections of ferroptotic inhibitors in OA treatment [76,99]. Rabbits are also relatively easy to use and have been utilized to study chemical effects and for models of ACL and meniscus surgery [98]. ACL-relevant animal models that somewhat resemble the human knee joint can also be used to simulate primary ACL repair [100]. Canine models are useful due to their anatomical and biomechanical similarities to humans and their ability to be examined using similar diagnostic procedures used in humans [98].

### 5.1. Canine

Canine models of hemarthrosis have yielded significant insights into *the* in vivo impact of blood injections in the knee [10,24,53,73,101]. The use of canines as an in vivo model for blood-induced joint damage has been supported by various publications including a 1999 study that evaluated the effects of injecting autologous blood into the right knees of beagles on days 0 and 2 of experimentation. The results of this study demonstrated a decrease in the number of proteoglycans in the cartilage matrix throughout the evaluation on days 4 and 16 [24]. The observed short-term effects include an increase in denatured collagen, inhibited proteoglycan synthesis, and inflamed synovial tissue on day 4. By day 16, the long-term effects included enhanced proteoglycan synthesis and cartilage-destructive properties in synovial tissue [24]. Overall, the exposure of the joint to blood for a short time period was shown to result in damaging effects on the cartilage matrix, chondrocyte metabolic activity, and the synovium [24].

In terms of ACL repair, researchers have also compared the efficacy of partial transection versus synovial debridement (i.e., removal of the sheath) of the cranial cruciate ligament (CCL) in canine models [102,103]. Differential changes to the synovium and cartilage of ACL transection in canine models have been directly linked to surgical techniques that induce bleeding [100]. Extensive ligament research has been conducted using canine models but many repair procedures that are effective in humans do not translate as well to canines, diminishing their clinical relevance [104]. In support of strategies to mitigate joint bleeding impacts, Myers and co-workers found that 69% of dogs showed synovitis in the OA knee 10 weeks after anterior cruciate ligament transection (ACLT). When hemostasis was maintained via electrocautery and irrigation before joint closure, synovitis was present in only 24% of the OA knees 10 weeks after ACLT (*p* < 0.01). A total of 75% of synovial samples exhibited iron deposits after routine ACLT but in only 6% (*p* < 0.001) of the hemostasis-controlled group [103]. We speculate that together with reports showing that immediate repair of ACL transection yields better functional outcomes than delayed (2 or 6 weeks) repair, joint bleeding effects may contribute to the development of OA despite ACL reconstruction [119,120].

Canine animal studies can also be employed to test the efficacy of various anti-ferroptosis agents at ameliorating blood-induced synovial joint injury and damage [75,105,106]. Once the in vivo efficacy of anti-ferroptosis agents is proven with an administration protocol that maximizes the chance of drug bioavailability, future studies can refine the drug delivery protocol, including the incorporation of sustained intra-articular delivery, similar to techniques used to deliver dexamethasone in a canine model of cartilage repair or conjugation with carriers that prolong residence time [73,75,121]. Preliminary data using small animal model findings provide proof-of-concept data to warrant studies using large preclinical animal models, such as the canine model, where established models of hemophilic arthrosis and PTOA can be adopted [10,67,96,97,107–109,122].

Studies have shown success replicating hemarthrosis, with one study doing so by using canine joints to determine whether intra-articular injection of coagulated or anticoagulated blood was more destructive to the joint [107]. Though this group had access to human cartilage explants, the canine models were advantageous because their knees could be used for direct injection and the effects could be studied in a large animal model subject to daily physical activity and a controlled diet. Post-mortem cartilage explants from these canines were tested for proteoglycan synthesis, synovial inflammation, and the catabolic effects of the inflammation. The canine knee models demonstrated that knees injected with coagulated blood led to a higher amount of proteoglycan synthesis than in the groups injected with anticoagulated blood, a result of ineffective cartilage repair activity [107]. Additionally, when compared to controls with saline injections, macroscopic and histological results showed that the coagulated blood group experienced a much higher percentage increase in inflammation [107]. In this acute hemarthrosis protocol, dogs were subjected to four blood injections in four successive days, which is repeated after two weeks. This led to irreversible joint injury that is seen 4 days post blood exposure, and which progresses to degenerative changes reminiscent of OA seen in a timeframe of 14 weeks [10,73]. Overall, this study showed that anticoagulation limits the damage caused by blood [107]. However, it is important to note that though this avenue is not fitting for hemophilic patients due to their need for clotting factors to stop bleeding, it sheds light on the role blood plays in joint damage [107].

In addition to pharmacological agents, canine models are also useful for studying tissue engineering methods of healing. In a 2008 study, the stifle joints of canines were used as models of osteochondrosis (OC). Autografts were applied to the affected area using the osteochondral autograft transfer system (OATS) procedure. After 6–8 weeks, the dogs were significantly less lame, using a lameness index [108]. Upon 6–15-month post-surgery clinical evaluation, these in vivo studies provided evidence for the functionality and feasibility of using osteochondral grafts. The grafts demonstrated the integration of the bone and the maintenance and restoration of sections of the articular cartilage [108].

### 5.2. Lapine/Rabbit

Rabbit models are often utilized in musculoskeletal research due to being the largest “small” animal model and due to their cost-effectiveness [110]. One commonly used rabbit study induces joint bleeding by drilling bone holes in the femoral notch, where intra-articular bone injury reproducibly creates early joint inflammation with some chronic synovial changes and progressive cartilage damage consistent with OA in adult rabbits in 9 weeks [74,81,110,111]. This Huebner study is the first to create a surgical model of OA in which the joint was not destabilized and its biomechanics were not affected [74]. This intra-articular bone injury creates reproducible early joint inflammation with some chronic synovial changes and progressive cartilage damage consistent with OA in 9 weeks that further degenerates to 52 weeks [81,110,111]. The drill holes in experimental animals progressed from early callus and fibrocartilaginous callus to bone in 52 weeks.

The past literature has shown rabbit studies highlighting the impact of acute hemarthrosis on the histological, biomechanical, and biochemical characteristics of intact ACL [112]. Joint bleeding prior to or due to ACLR has been suggested to play a role in the clinical finding that patients ± ACLR develop OA [119,120]. In a separate much earlier study from the Woo group, daily blood injection in the rabbit knee did not show a change to ACL architecture or structure-function [112]. Conversely, rabbit models have been proven to demonstrate a molecular response, such as mRNA expression of pathological markers, that results from induced hemarthrosis to the meniscus. This study followed up with a single intra-articular injection of dexamethasone (DEX) using the method developed by Huebner [81]. DEX administration led to events that are typically seen following induced hemarthrosis, including a decrease in key catabolic markers, normalization of the gene expression of inflammatory mediators, and suppression of the mRNA expression of molecules in the autophagy pathway [81].

Another study that models OA without joint instability was conducted by injection of interleukin-17 (IL-17) into the articular cavity of the knee. Blood exposure and cartilage degradation are commonly associated with increases in IL-1*β*, TNF-*α*, IL-6, and IL-17 levels. While IL-1*β* is one of the most recognized pro-inflammatory cytokines, studies have shown that IL-17 similarly induces the degradation of the cartilage extracellular matrix [113]. Therefore, IL-17 injections were administered once every 2 days, for a total of 3 injections. Knees that were injected with IL-17 varied in concentrations and were compared with a preceding method of inducing OA, the Hulth method [113]. This study resulted in an identification of an optimal concentration of IL-17 that does not result in the joint destabilization caused by the Hulth method. The injection of 10 ng/mL of IL-17 led to morphological changes that are typical of late-stage OA. Additionally, the model led to an increase in pro-inflammatory factors that promote synovial hyperplasia and fibroblasts that lead to angiogenesis [113]. Overall, the similarity in the enzyme expression, radiological evaluations, and histological scores demonstrate that this group’s dosage of IL-17 in a rabbit model produces similar effects to posttraumatic OA.

Although Fer-1 has been used in mice/rat in vivo studies, the application to the rabbit is novel and represents a critical step toward translation to large preclinical animal models. The scale of the rabbit knee joint (and synovial fluid cavity volume) is more practical than the mouse or rat knee joint for intra-articular injections of therapeutic agents. The larger anatomy of the synovial joint ensures intra-articular placement and can better tolerate injections that induce bleeding and local joint damage that may impact the outcome of the proposed studies aimed at understanding joint bleeding-induced arthropathy.

The adoption of a small preclinical Lapine model validates the assessment of hemarthrosis changes to the knee joint and introduces the opportunity to assess the ability of ferrostatins to slow progression/prevent degenerative synovial joint changes induced by joint bleeding [74].

### 5.3. Rodent

Ferrostatin studies have been performed using rodents; the current studies represent a translational advance with preclinical and clinical relevance [11,59,76,114]. These small animal model findings provide proof-of-concept data to warrant studies using large preclinical animal models, such as the canine model, where established models of hemophilic arthrosis and PTOA can be adopted [10,67,73,96,107–109,123]. In most rodent studies, ferroptosis was triggered by blood and ROS as a result of OA biomechanical instability. Although Fer-1 mitigated OA changes in rodents, we are unsure if it would be successful in treating blood-induced injury. Nevertheless, these studies provide an in vivo experimental drug protocol that can be used to investigate the effects of blood exposure.

While rodents do not accurately recapitulate the human anatomy, they are useful in determining mechanisms of pathology, as mentioned above [96]. In a 2019 study, mice models were used to discover that ferroptosis is a mechanism of cardiomyopathy induced by doxorubicin and ischemia/reperfusion [59]. Using mice served as a useful disease model to study the efficacy of cardiomyopathy-inducing drugs and to test the therapeutic effect of inhibitors on a diseased subject. With this information, the group discovered that ferroptosis in cardiac injury is a result of the upregulation of heme oxygenase Hmox1 that causes the heme degradation and release of free iron that leads to ferroptosis [59]. Importantly, using small animal models like rodents makes it easier to test different factors and environments that could promote therapeutic effects. In addition to identifying mechanisms of cardiomyopathy, this group tested the role of low-iron diets and certain inhibitors in reducing the risk of death, paving the way for future studies to utilize these findings [59].

In another study, rats were used to further investigate the mechanisms of ferroptosis in an in vivo model. In vitro studies utilized IL-1β to simulate inflammatory conditions, and iron overload was replicated using ferric ammonium citrate (FAC) in order to validate these reagents as inducers of ferroptosis [76]. Their studies demonstrated that these two reagents did cause changes in the protein expression of markers indicative of ferroptosis (GPX4, ACSL4, P53, SLC7A11) [76]. After in vitro validation testing, the inhibitory effects of Fer-1 were tested in a mouse OA model. The OA model was established in the mice by performing destabilized medial meniscus (DMM). With unaffected and DMM mice, the group tested the effects with and without Fer-1 injections. Safranin-O staining revealed that the injection of 1 mg/kg Fer-1 lessened cartilage degeneration. Additionally, GPX4 (a key regulator of ferroptosis) staining showed that the injection of Fer-1 increased the amount of GPX4-positive cells in the DMM groups [76]. Overall, this study proved that Fer-1 exhibits anti-OA effects in vivo.

## 6. Strategies to Promote Joint Repair

PTOA is thought to be exacerbated by hemarthrosis as it reduces joint lubrication, causes synovitis, and indirectly damages cartilage [124]. Surgical intervention is prescribed based on the patient’s activity level, functional demand on the knee, occupation, and presence of an associated injury in the knee joint, where surgery is chosen in most active, younger patients [125]. However, despite wound drainage after surgery, bleeding in the patient’s surgical area cannot be completely discharged, and some bleeding remains in the joint cavity and tissue space and is gradually absorbed, organized, and exuded [7]. The concentration of blood in the synovial cavity reaches nearly 100% volume/volume (*v*/*v*) during hemarthrosis episodes as a result of the small, almost negligible volume of intra-articular synovial fluid (0.5–4.0 mL) for normal human knees and has been confirmed in in vivo rat and rabbit models [10,126]. Reported studies have shown that a minimum concentration of 10% *v*/*v* blood for 2 days induces long-lasting detrimental changes to human cartilage in vitro (i.e., inhibition of proteoglycan synthesis) while concentrations exceeding 50% *v*/*v* blood have been clinically shown in traumatic joint bleeds [72]. Therefore, research is needed on blood expulsion from the diarthrodial joint to promote joint healing. Current strategies directly target surgical improvements and involve enhancing surgical procedures. Alternatively, a therapeutic approach might be more effective but no DMOAD currently exists and Fer-1 or a combination of other drugs (anti-apoptosis or other cell death mechanisms) may need to be investigated to effectively prevent subsequent sequelae leading to OA.

### 6.1. Surgical Intervention & Current Repair Techniques

Evacuation of Blood Post-Injury (or Surgery)

To curtail the deleterious effects of blood, some clinicians have advocated for the aspiration of blood from the joint post-injury and prior to surgical closing [5,6]. This strategy may be undermined, however, by continued joint bleeding, or hidden blood loss and by erythrophagocytosis by synovial macrophages, which can lead to synovial iron deposition (hemosiderosis), arising from iron overload induced by heme, a red blood cell (RBC) breakdown product [6,7,47,48]. Hemosiderosis may not be reversible, serving as a permanent trigger for immune and synovial cells to induce the production of IL-1 and TNF as well as fibroblast proliferation leading to tissue hyperplasia [49,52].

Interestingly, however, mesenchymal stem cells (MSCs) have been derived from hemarthrosis and have shown greater viability than those from remnant tissue [127]. These hemarthrosis-derived MSCs may offer further tissue engineering methods to alleviate the detrimental effects of hemarthrosis [128]. These MSCs can be obtained in sufficient quantity during the evacuation of blood from the joint, will likely not promote an immunogenic response, and have demonstrated multipotency. Studies have also shown that bone-derived MSCs are mobilized and can migrate toward the injured joint but therapeutic strategies have not yet taken advantage of MSCs [84].

### 6.2. Platelet-Rich Plasma (PRP)

Platelet-rich plasma (PRP) is a regenerative therapy popular in musculoskeletal medicine [129]. PRP is an autologous enrichment of blood plasma with platelets to a concentration above normal blood levels [130]. PRP’s therapeutic potential stems from platelets’ capacity to supply and release growth factors and cytokines; the high concentration of platelets in PRP offers supraphysiologic amounts of these healing molecules to promote the repair of tissue [131].

A study of the fusion of extracellular matrix (ECM) proteins and blood into a composite injection was shown to reduce PTOA in mice following ACLR, possibly due to the prolonged release of platelet-associated growth factors [17,115,132]. While ECM proteins have been used to induce rheumatoid arthritis in animal models, the ECM scaffold combined with the release of platelets is thought to protect the matrix within the joint, treating PTOA [133].

PRP has been shown to improve bone–patellar tendon–bone allografts but has not been shown to improve the mechanical function or clinical properties of allografts after 24 months [129]. The efficacy of PRP application in graft-to-bone integration has varied but there has been some evidence of benefits like enhanced healing, reduced edema, increased vascularity at the bone–graft interface, and a 48% decrease in the amount of time for the graft to achieve a “ligamentous-like” MRI signal [129]. Due to the conflicting nature of PRP’s role in ACL healing, there appears to be insufficient evidence to suggest that the clinical outcomes of surgery are improved by the use of PRP [134]. Further research and standardized studies into PRP’s role in ACL graft maturation and joint healing will improve our understanding of PRP’s regenerative properties.

## 7. Potential Therapeutics

Fer-1 is a potent and selective inhibitor of ferroptosis that acts by suppressing lipid peroxidation through lipophilic peroxyl radical trapping activity [11]. However, the absorption, distribution, metabolism, and excretion (ADME) properties of Fer-1 make it challenging to use in animal models. Alternative drug-like ferrostatins with ADME properties suitable for in vivo use still need to be studied. The usage of ferroptotic therapeutic treatment would represent a significant advance in the understanding of blood damage and ferroptosis. With regard to the in vivo administration of anti-ferroptosis agents, there are promising findings demonstrating that biweekly injections of Fer-1 can inhibit cartilage degenerative changes in posttraumatic OA (PTOA) in mice, and a single injection of Fer-1 can inhibit neuronal degeneration after an intracerebral hemorrhage in the mouse [76,116,117]. This PTOA is due to surgically induced biomechanical knee instability because they cut the meniscus. It has not yet been demonstrated if Fer-1 can mitigate the deleterious effects of joint bleeding but this would represent a potential candidate. Together, these studies suggest that Fer-1 by itself or in combination with other drugs could serve as a DMOAD.

With an agent whose bioavailability has been optimized via drug design for enhanced ADME properties, a newly devised injection protocol is needed to counter their short half-life, due to their rapid clearance rate from the joint space [60]. In general, soluble materials have an intra-articular dwell time measured only in hours [135]. For context, the residence time of blood in the rabbit joint is at least 4 h longer with synovial inflammation [70]. The multiple IA injection strategy supports the use of the rabbit rather than much smaller mice/rats, where repeated injections could introduce additional bleeding that could create confounding synovial joint injury and pathology [118]. Additional studies have shown that the fully reduced forms of Vitamin K have strong anti-ferroptotic functions and the potential to protect cells from lipid peroxidation [136]. This could serve as an alternative inhibitor of ferroptosis.

Another alternate ferroptotic inhibitor is protopine (PTP). An isoquinoline alkaloid, PTP is an active compound in *Corydalis yanhusuo* W. T. Wang that has several anti-inflammatory and antioxidant properties [137–139]. PTP mitigates cell injury by down-regulating the expression of NLRP3 and NF-κB signaling pathways, thereby diminishing inflammation and oxidative stress [137]. In a mouse ligament study, PTP was shown to ameliorate ferroptosis after ACL transection surgery, improving the protection of chondrocytes [140]. These findings prove that ferroptotic inhibitors offer a promising therapeutic in mitigating the impact of blood in synovial joint tissues.

## 8. Conclusions

This review highlights the prevalence of blood-induced arthropathies as a result of traumatic knee injuries or congenital disorders. A better understanding of the impact of blood on synovial joint tissues will support research efforts aimed at mitigating blood-induced joint damage, such as the development of prophylactic treatments of disease-borne, trauma- and iatrogenic-induced joint bleeding. Toward these efforts, biofidelic in vitro models can offer well-defined experimental platforms to complement in vivo models [141]. Co-culture models that include the synovium, which envelops the joint, with other tissue(s) in the knee may provide additional information regarding tissue crosstalk and help to identify complementary therapeutic targets to mitigate the contribution of joint bleeding to the development of arthropathy [79]. The incorporation of mechanical loading that may exacerbate joint degeneration, by providing physical stimuli or enhanced solute transport, may further provide a more physiologic context to study blood and synovial joint tissue interactions [142–144]. Blood-injured cartilage, for example, has been reported to be less capable to withstand loading in vivo [53,55]. Additionally, the study of blood and its behavior in synovial fluid may also be an important consideration [77]. These culture models may also permit the optimization of adjunctive therapies, such as corticosteroids, or the application of pulsed electromagnetic fields (PEMFs) [105,106,145].

Our findings, from investigating blood co-culture with chondrocytes, lead us to suggest that ferroptosis plays a crucial role in HA development and could result in adverse effects on joint tissues [14]. While ferroptosis has been shown to contribute to blood-induced cartilage degenerative changes, its role in modulating tissue changes to the synovium, meniscus, ligament, and bone remains to be determined. In this context, as previously mentioned, Fer-1 is a potent and selective inhibitor of ferroptosis that acts by suppressing lipid peroxidation through lipophilic peroxyl radical trapping activity [11]. However, Fer-1’s ADME properties make it challenging to use in animal models. New Fer-1 analogs, with improved potency and metabolic and plasma stability, would lay the foundation for a new therapeutic approach to treating these patients and other related blood-based conditions involving ferroptosis.

Current research investigating the role of the interaction of blood with synovial tissues is primarily focused on cartilage and chondrocytes as cartilage degeneration is the leading comorbidity. Additional research is necessary to understand the contribution of joint bleeding to changes in the maintenance and repair capacity of other synovial joint tissues like the synovium, meniscus, bone, and ligament. Our data suggest that ferroptosis may represent a therapeutic target with regard to joint degeneration associated with joint bleeding. The application of experimental in vitro and in vivo models of hemarthrosis, as reviewed herein, may help to inform the development of therapeutic treatments for synovial joint bleeding.

## Figures and Tables

**Table 1.** In vitro models for blood-induced arthropathy studies.

	Reference	Experimental Design	Research Findings
CARTILAGE	Convery et al., 1976 [55]	Biochemical and biomechanical analyses of mongrel dogs subject to continuous hemarthrosis [55]	Significant decrease in GAG content observed as early as 4 weeks. Total collagen content was not significantly affected until after 12 and 16 weeks. Experimental cartilage lost significant amount of mechanical integrity as a result of continuous hemarthrosis [55].
	Roosendaal et al., 1999 [52]	Co-culture of human cartilage tissue with RBC or mononuclear cells (MNC) + RBC followed by a 12-day recovery period [52]	Acute MNC + RBC exposure resulted in prolonged dose-dependent inhibition of proteoglycan synthesis and chondrocyte death [52].
	Hooiveld et al., 2003 [9]	Co-culture of human cartilage tissue with whole blood, mononuclear cells + RBC, or caspase inhibitors followed by a 12-day recovery period [9]	After 4-day exposure of blood treatment, apoptosis occurred in chondrocytes. Caspase inhibitors partially restored proteoglycan synthesis after blood exposure [9]
	Hooiveld et al., 2003 [61]	Co-culture of human cartilage tissue with RBC, mononuclear cells + RBC, CD14+ cells + RBC, or lysed RBC with IL-1*β* followed by a 12-day recovery period [61]	Lysed RBC + IL-1*β* produced by monocytes inhibited proteoglycan synthesis and increased production of hydrogen peroxide. Hemoglobin-derived iron + hydrogen peroxide resulted in formation of hydroxyl radicals that lead to further chondrocyte damage. DMSO and hydroxyl radicals can restore cartilage proteoglycan synthesis [61].
	Jansen et al., 2007 [72]	Healthy human articular cartilage explants cultured in the presence or absence of varying blood concentrations for different time intervals followed by a 12-day recovery period after withdrawal of blood [72]	50% *v*/*v* blood exposure to cartilage led to detrimental effects independent of time. Blood concentration-dependent effects were observed and after 2 days of exposure of at least 10% *v*/*v*, effects were irreversible [72].
	van Meegeren et al., 2012 [73]	Human cartilage explants exposed to coagulating and anticoagulated blood, plasma and serum [73]	Exposure of coagulating blood resulted in more damage than anticoagulated blood. In absence of mononuclear cells and RBC, plasma and serum exposure did not alter cartilage matrix turnover [73].
	Huebner et al., 2013 [74]	Gene expression analyses of cartilage from a rabbit bone drill PTOA model [74]	Upregulation of cartilage degradation markers including TGF*β* and MMP13, suggesting that intra-articular bone injury contributes to progressive cartilage damage consistent with OA in adult rabbits [74].
	van Meegeren et al., 2013 [7]	Evaluation of biochemical properties of canine explants comparing effects of acute blood exposure vs. intermittent intra-articular blood injections [7]	Explants exposed to acute and micro bleeds saw increased proteoglycan synthesis rates. Release of newly formed GAGs was significantly increased following acute blood exposure [7]
	Bajpayee et al., 2017 [75]	Gene expression and biochemical analysis of rabbit cartilage following intra-articular DEX administration in a rabbit ACL transection model [75]	No significant differences observed in collagen content between ACLT and control. Significant decrease in GAG content observed in cartilage of ACLT in comparison to control. Avidin-DEX treatment suppressed catabolic gene expression and joint swelling significantly more than free DEX treatment [75].
	Yao et al., 2020 [76]	Simulate inflammation and iron overload via administration of IL-1*β* and ferric ammonium citrate [76]	Under inflammation and iron overload, chondrocytes undergo ferroptosis. Ferroptotic induction via erastin caused increased cartilage degradation enzymes and decreased collagen II expression [76].
	Lee et al., 2023 [11]	Co-culture of human chondrocyte-based tissue engineered cartilage and human chondrocyte monolayer with intact RBC and RBC lysates [11]	Markers of tissue breakdown were observed in cartilage constructs without parallel losses in DNA. Dose-dependent loss of viability observed in chondrocyte monolayers, with greater toxicity observed with lysates; intact RBCs induced changes to lipid profiles and upregulated highly oxidizable fatty acids; RBC lysates induced cell death via ferroptosis [11]
SYNOVIUM	Muirden et al., 1967 [47]	Assess ferritin formation by synovial cells exposed to hemoglobin [47]	Findings suggest that synovial cells release iron from hemoglobin thereby synthesizing apoferritin without other cell intervention [47]
	Roosendaal et al., 1999 [24]	Analysis of catabolic activity of supernatants of synovial tissue after intraarticular blood exposure in canine model [24]	After 4 days of blood exposure, synovial tissue had no significant catabolic activity. After 16 days, synovial tissue had significant cartilage destructive properties. The supernatant of synovial tissue decreased total proteoglycan content and lowered proteoglycan synthesis [24].
	Hooiveld et al., 2004 [69]	Biochemical analysis of synovial fluid of PTOA guinea pig model [69]	Stromal cell-derived factor-1 (SDF-1) and MMP13 synovial fluid concentration were statistically similar between idiopathic and PTOA groups suggesting that other inflammatory factors may be involved [69]
	McCarty et al., 2011 [77]	Characterize composition, coagulation and mechanical properties of blood and synovial fluid mixtures [77]	Dilution of blood with SF decreased mechanical stiffness of the final clot structure, altered coagulation torque profile over time, and increased fluid permeability. In comparison to control, SF had a lesser effect on mechanical properties of clot possibly due to presence of HA [77]
	Ashraf et al., 2011 [78]	Measure synovial inflammation and angiogenesis as a result of meniscal transection in a rat OA model [78]	Synovial inflammation increased 24 h after drug-induced synovitis. Increase of macrophage infiltration and endothelial cell proliferation confirmed synovitis and angiogenesis after meniscal transection. Anti-inflammatory drugs inhibited synovitis and synovial angiogenesis [78].
	Stefani et al., 2019 [79]	Investigation of solute transport measures in bovine tissue-engineered synovium model [79]	NO and hyaluronic acid (HA) secretion of engineered synovium was similar to native synovial tissue. IL-treated engineered synovium displayed characteristics of human OA synovium [79].
	Machado et al., 2019 [80]	Compare effects of blood-derived products and sodium hyaluronate on equine synovial fluid cells from osteochondrotic joint [80]	All treatments decreased production of reactive oxygen species. PRP increased prostaglandin (PG) E2 concentrations and PRP, APP, and IRAP increased IL-1 receptor antagonist proteins [80]
MENISCUS	Heard et al., 2019 [81]	Acute controlled response of meniscal cell to blood and blood + DEX [81]	Autologous blood exposure did not affect meniscal cell viability but blood + DEX treatment reduced cell viability suggesting that meniscal cells may be susceptible to glucocorticoid-mediated apoptosis. [81]
	Betsch et al., 2024 [82]	Porcine explants and primary meniscus cells exposed to various concentrations of whole blood for 3 days to simulate injury [82]	Acute whole blood exposure increased MMP activity. Blood-derived mononuclear leukocytes increased NO release and MMP activity but decreased GAG content suggesting that they activate catabolic pathways that can lead to meniscal degradation. Isolated intact RBC did not affect meniscus catabolism. [82]
BONE	Niikura et al., 2005 [83]	Assess potential of hemarthrosis-derived cells to differentiate into osteoblast-like cells via gene expression and osteoblastic markers [83]	Osteoblastic gene markers significantly increased in hemarthrosis-derived cells in comparison to control and cell morphology was cuboidal shaped. Human hemarthrosis-derived cells contain osteoprogenitor cells and can be used for bone regeneration [83]
	Maerz et al., 2017 [84]	Rats were subjected to noninvasive ACL rupture. Whole blood MSC concentration was assessed using flow cytometry and synovial fluid and serum were assayed for stromal cell-derived factor (SDF-1*α*) [84]	Following ACL rupture, there was a significant increase in bone marrow-derived MSC concentration and SDF-1*α*. Cell tracking indicated active recruitment of MSCs to the injured joint. [84]
ACL	Jacobson et al., 2008 [14]	Measure effect of platelets and erythrocytes on cytokine release and scaffold contraction in a 3D fibroblast model [14]	Platelet concentration significantly affects fibroblast proliferation, cytokine release, and gel contraction [14]
	Harrison et al., 2011 [13]	Analyze effect of erythrocyte concentration on wound healing capacity of an ACL fibroblast collagen scaffold [13]	Greater scaffold contraction and fibroblast proliferation of samples with RBC concentrations lower than that in whole blood. Increasing RBC concentration over amount in whole blood stimulates fibroblast collagen production and decreases scaffold contraction. [13]
	Yoshida et al., 2013 [85]	Examine effect of peripheral blood mononuclear cells (PBMCs) on 3D ACL fibroblast collagen scaffold [85]	Co-culture of PBMCs and ACL fibroblasts without presence of platelets had no effect. PBMCs + platelet exposure led to an increase in collagen gene and protein expression as well as increased IL-6 expression. [85]

**Table 2.** In vivo models for blood-induced arthropathy studies.

Species	Advantages	Disadvantages	Applications
Canine 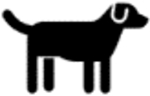 Ref: [7,24,53,55,67,73,95,99,101–109]	Able to use similar procedures and assessments to those used in human models Similar disease progression to humans Post-operative assessments ate similar to human	High cost Ethical and public considerations	Hintological, biochemical and biomechanical evaluation Can be studied via MRI, CT, and radiography
Lapine 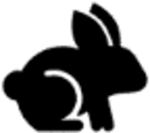 Ref: [70,74,81,94,110–113]	Availability Relatively low cost compared to larger animal models	Different disease progression timetine (faster cartilage regeneration) Postoperative management differs	Screening of treatments Study of disease mechanisms Screening of drugs, biologics and therapeutics Histological, bioclnemical and biomechanical evaluation
Rodent 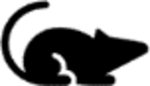 Ref: [59,76,93,98,114–118]	Availability Relatively easy screening of treatments Testing of xenogenic cells and tissues Low cost Space effective	Different disease progression timeline Small, thin joints and cartilage Postoperative manahement differs Gait and movement characteristics vary	Genetia manipulation Use of xenognnic cells and tissues Screening of drugs, biologics and therapeutics Study of disease mechanisms Histological, biochemical and biomechanical evaluation
